# Roles and mechanisms of biomechanical-biochemical coupling in pelvic organ prolapse

**DOI:** 10.3389/fmed.2024.1303044

**Published:** 2024-02-12

**Authors:** Huaye Wu, Ling Zhang, Li He, Wenyi Lin, Bo Yu, Xia Yu, Yonghong Lin

**Affiliations:** ^1^Department of Obstetrics and Gynecology, Chengdu Women's and Children's Central Hospital, School of Medicine, University of Electronic Science and Technology of China, Chengdu, Sichuan, China; ^2^Department of Medical Pathology, Chengdu Women's and Children's Central Hospital, School of Medicine, University of Electronic Science and Technology of China, Chengdu, Sichuan, China; ^3^Department of Clinical Laboratory, Chengdu Women's and Children's Central Hospital, School of Medicine, University of Electronic Science and Technology of China, Chengdu, Sichuan, China

**Keywords:** pelvic organ prolapse, fibroblasts, biomechanical-biochemical coupling, extracellular matrix, cytoskeleton

## Abstract

Pelvic organ prolapse (POP) is a significant contributor to hysterectomy among middle-aged and elderly women. However, there are challenges in terms of dedicated pharmaceutical solutions and targeted interventions for POP. The primary characteristics of POP include compromised mechanical properties of uterine ligaments and dysfunction within the vaginal support structure, often resulting from delivery-related injuries. Fibroblasts secrete extracellular matrix, which, along with the cytoskeleton, forms the structural foundation that ensures proper biomechanical function of the fascial system. This system is crucial for maintaining the anatomical position of each pelvic floor organ. By systematically exploring the roles and mechanisms of biomechanical-biochemical transformations in POP, we can understand the impact of forces on the injury and repair of these organs. A comprehensive analysis of the literature revealed that the extracellular matrix produced by fibroblasts, as well as their cytoskeleton, undergoes alterations in patient tissues and cellular models of POP. Additionally, various signaling pathways, including TGF-β1/Smad, Gpx1, PI3K/AKT, p38/MAPK, and Nr4a1, are implicated in the biomechanical-biochemical interplay of fibroblasts. This systematic review of the biomechanical-biochemical interplay in fibroblasts in POP not only enhances our understanding of its underlying causes but also establishes a theoretical foundation for future clinical interventions.

## 1 Introduction

Pelvic organ prolapse (POP) is a health problem for women in which the pelvic organs are displaced inside or outside the vagina. This condition is caused by weakness of the pelvic floor muscles or ligaments that results in herniation of the vagina and uterus and prolapse (1), leading to bladder and sexual dysfunction (2). POP seriously affects women’s physical and mental well-being and quality of life. Global burden of disease is relatively high, there were 13 million incident cases of POP in 2019, with an age-standardized incidence rate of 316.19 per 100,000 population at the global level (3). A large-scale population-based cross-sectional study of adult women in China found that the symptomatic prevalence of POP was 9.6% (4) and the incidence approached 50% in middle-aged women, making POP one of the leading causes of hysterectomy in middle-aged women. The latest start of attention and research on POP has resulted in a lack of understanding of its pathogenesis and a lack of development of effective targeted drugs. Although many surgical treatments can achieve ideal anatomical repositioning at the cost of uterine resection, they still do not ensure functional recovery and symptomatic improvements. Thus, the lifetime risk of undergoing surgery for prolapse is 20% (5) and the rate of reoperation was 26.9% of Danish women aged between 18 and 49 years of age at primary surgery (6). The age groups with the highest incidence rates are also on a downward trend (3).

The strongest risk factors for POP are pregnancy and vaginal delivery (4, 7–9). A systematic review (7) showed that the incidence of the first vaginal and forceps delivery contributed the most to POP. A complete cesarean section protected against POP symptoms and clinical symptoms, and there was no risk compared to that in nulliparous women (9). Moreover, women with 1 child were 4 times more likely and women with 2 children were 8.4 times more likely to experience POP that required hospital admission (10). For these reasons, vaginal delivery has been shown to be the most important independent predisposing factor for POP. There have been some theories suggesting that the processes of pregnancy and childbirth releases tremendous abdominal pressure on the pelvic floor. Additionally, it has been proposed that this force is amplified in cases of multiple births and that persistent obesity can increase pressure on the abdomen (11). Impaired mechanics of the uterosacral ligament (USL) due to long-term forces is a key tissue in the pathogenesis of POP (12). Level I support includes the USL complex, which extends from the cervix to the sides of the uterus where it connects to the sacral surface; the USL plays a crucial role in supporting the uterus and vagina (13, 14). However, the structural integrity of the USL can be compromised over time due to repeated stretching caused by factors such as pregnancy and standing postures. This weakening of the ligament can ultimately result in clinical prolapse. In addition, the vagina is extremely important to the pelvic floor and serves as an important support structure for the pelvic floor. Overall, numerous studies have shown that the USL and anterior vaginal wall undergo biomechanical stimuli, which activat signal pathways through a series of biochemical reactions, induce structural damage of connective tissues, leading to dysfunction. The ultimate expression is a decrease in the quality of mechanical rigidity and stiffness as well as pelvic floor dysfunction, leading to the occurrence of POP. Thus, understanding the molecular mechanisms of biomechanical and biochemical coupling in fibroblasts, which are the basic components of POP, is important for the prevention and treatment of POP.

## 2 POP and biomechanics

### 2.1 POP biomechanical features

The pelvic floor consists of highly supportive connective tissues, all of which are viscoelastic materials and whose mechanical properties are critical to the pelvic floor as a whole. In 2002 (15), a study reported an increase in the modulus of elasticity of the anterior vaginal wall in postmenopausal compared to premenopausal POP patients for the first time, identifying an increase in strain and a decrease in stiffness in response to the same force in menopausal POP. This finding indicates decreased vaginal elasticity, which is possibly related to aging. However, a case–control study showed that the vaginal wall elastic modulus increased significantly in postmenopausal women, which affirmed that vaginal tissue is less elastic, and stiffness increased in POP group. Ultimately, decreased elasticity leads to decreased mechanical properties of the vaginal wall even though controlling for confounders such as age, body mass index and parity (16). These studies suggest that a reduction in pelvic biomechanical performance is a direct cause of POP. Additionally, the normal USL has been shown to support more than 17 kg (17) in mechanical experiments *in vitro*. In contrast, the flexibility of the USL was significantly reduced by a factor of 4 in patients with POP (18), and there was significant thinning during transvaginal delivery, weakened tissue mechanics, and reduced elasticity leading to the development of POP. The decrease in tissue biomechanical properties and dysfunction are closely related to the fate of the pelvic floor, and histological observations indicate that prolapse induces atrophy of the muscularis. Pathologic findings show that prolapse induces a decrease in each layer of the vaginal wall and atrophy of the muscularis propria (19). Fibroblasts produce extracellular matrix (ECM) and are the major cells in the pelvic floor. The ECM is a complex structure primarily composed of collagen and elastin fibers. It plays a crucial role in providing the pelvic floor with the necessary tensile strength and resistance against external forces. Additionally, the ECM connects the supportive tissues, facilitating the transmission and distribution of these external forces throughout the pelvic floor (20). Proteomics and transcriptomics show that the USL is continuously remodeled through a decrease in collagen synthesis and an increase in collagen type I (COL1) and type III (COL3) degradation in POP patients, which changes the microenvironment of tissue mechanics (21). Current research indicates that dysfunction of ECM in vaginal wall and USL decreases biomechanics, which is a major factor in POP.

### 2.2 Structural basis of biomechanics in POP

The phenotypic alterations observed in prolapsed tissue at the macroscopic level *in vivo* are indicative of underlying cellular changes, which can be attributed to increased mechanical stretching. Fibroblasts, the primary cellular component of the pelvic floor, exhibit modified cellular attributes *in vitro*, which influences their dynamic response to external mechanical stimuli. The ECM is critical for matrix mechanics and sensing cell stiffness. Lysyl oxidase (LOX), lysyl oxidase-like 1–4 (LOXL 1–4), a disintegrin-like and metalloproteinase with thrombospondin type 1 motifs 2 (ADAMTS2), and bone morphogenetic protein 1 (BMP1) play crucial roles in maintaining collagen stability and facilitating the formation of procollagen (22). Studies have demonstrated that human vaginal fibroblasts (HVFs) obtained from POP patients exhibit upregulated matrix metalloproteinases 3 and 7 (MMP3, MMP7), which facilitate collagen degradation and downregulate the genes responsible for collagen and elastin fiber synthesis and maturation (LOX, LOXL1-LOXL3, BMP1, and ADAMTS2) (23). Consequently, this leads to a reduction in collagen synthesis and production within the pelvic floor tissue of women with POP. Furthermore, significant differences were observed in the cell-matrix adhesion molecules LAMB1 and LAMB3 (basement membrane) (23). The adhesion of POP-HVFs to the ECM was decreased, resulting in impaired tissue morphology at the microstructural level. In terms of cellular morphology, a separate study revealed severe disruption of fibroblast cytoskeletal actin filaments in fibroblasts from POP patients, suggesting a close association between abnormalities in this cellular support protein and external mechanical stretching (24). Besides, increasing evidence suggests that the vaginal microbiome and the immune system are closely linked to maintain a healthy vaginal environment and lower genitourinary health (25). That equally worthy of researchers’ attention.

## 3 Biomechanical-biochemical coupling of fibroblasts in POP

### 3.1 Cell-mediated biochemical remodeling of the ECM

Li et al. (19) demonstrated that fibroblasts were the most abundant cells in the vaginal wall, and these cells make up an impressive 55.49% of the vaginal wall. Consistent with previous studies, fibroblasts secrete and synthesize ECM and are regulated by several factors to maintain the structural integrity and functionality of the pelvic organ. (1) ECM homeostasis is dependent on the expression level of MMPs, a highly conserved family of endonuclease hydrolases that degrade ECM proteins and the pericellular microenvironment, including collagen, elastin, and laminin. Notably, vaginal wall ECM is enriched in COL1 and COL3 to support the vaginal wall; collagen type I provides strength to the tissue, while COL3 provides elasticity. Multiple studies have shown that MMPs (MMP1-2, MMP8-10) are significantly increase in the HVFs of patients or healthy vaginas after stress stretching (26–29). This increase leads to a noticeable reduction in the levels of COL1 and COL3, which are essential components of the ECM. Notably, MMP2 and MMP9 have been extensively studied and consistently found to be highly expressed in pelvic floor tissues due to their pivotal role as primary proteases that negatively regulate the ECM. (2) Additionally, ECM homeostasis is dependent on the expression of metalloproteinase inhibitors. Tissue inhibitors of metalloproteinases (TIMPs) are endogenous inhibitors of MMPs, which are enzymes that play central roles in the degradation of ECM components. Among them, TIMP2 can directly bind to MMP2 and is expressed at 10-fold higher levels than TIMP1 and TIMP3 (30, 31). Intriguingly, in the context of mechanical stretching, the expression of MMPs and the enzymatic activity of MMP2 were significantly increased in the HVFs of patients with POP. Conversely, the expression of TIMP2, which is a collagen inhibitor, was downregulated in comparison to that in healthy individuals with normal vaginal function (28). This downregulation of TIMP2 can have profound consequences on the anterior vaginal wall. When TIMP2 is inhibited, the degradation of ECM becomes less restrained. Consequently, collagen and adhesion proteins secreted by fibroblasts undergo rapid degradation, leading to the disruption of the delicate balance between the ECM and subsequent remodeling. The findings suggest a potential mechanism by which the excessive degradation of ECM components contributes to the development and progression of POP. It was also interesting that POP fibroblasts were more sensitive to mechanical forces and expressed higher levels of ECM-related regulatory proteins. The implication is that abnormal mechanical stress leads to the dysregulation of ECM homeostasis and promotes ECM reprogramming, leading to pelvic floor laxity. (3) Estrogen regulates ECM expression and components. A study by Zong et al. confirmed the increased mechanical sensitivity of POP-HVFs to 1 Hz cyclic loading for 72 h, increased induction of collagenase activity, and increased collagen degradation with increasing mechanical stretch force. Interestingly, estrogen only had a minimal inhibitory effect on mechanical damage (32). Thus, a decrease in estrogen accelerates mechanical damage, which may explain the clinical prevalence of POP in postmenopausal women. In 2018, poststretch estrogen intervention upregulated COLI in normal and POP cells, and the mechanical properties were consistent with the transcription of COL1, while COL3 remained unchanged, indicating an increase in tensile strength, which in turn affected the structure and function of COL1 in response to tensile loading, ultimately leading to abnormalities in the composition of the pelvic support connective tissue (29). (4) Other molecules, such as small proteoglycans are involved in POP. Decorin (DCN) and fibromodulin (FMO) are members of the small leucine-rich proteoglycan (SLRP) family that bind to different cell surface receptors and other ECM components to regulate the assembly of cellular collagen microfibrils. Recent studies have shown that the expression of DCN and FMO is downregulated in stretched POP-HVFs (29). According to Kufaishi et al. (29), the reduced expression of the enzymes LOX, LOXL1-2, and BMP1 was a result of exposure to mechanical stretch. This exposure led to a decrease in the cross-linking of collagen and elastin polymers, ultimately resulting in the weakening of connective tissue (28). However, it has been found that the ECM also influences cellular behavior, and the interaction between the two factors is complex (27). Dynamic reciprocity between the cell and its associated matrix is essential for maintaining tissue homeostasis and the dysregulation of ECM mechanical signaling. POP-HVFs were seeded in 6-well plates covered with type 1 collagen and cellular mechanical stimulation is subsequently loaded. The results found that with the increase of mechanical force, the effect of cellular mechanical response weakened. Then, HVFs delayed collagen contraction and downregulated MMP2 expression. Inversely, in noncoated collagen POP-HVFs, the gene expression of MMP2 and TIMP2 was upregulated, and the ECM was reduced. The functional characteristics of noncoated collagen POP-HVFs are mainly determined by the cellular surface matrix, indicating that the interaction between POP-HVFs and the matrix is compromised, leading to the inability of fibroblasts to respond promptly to mechanical stress signals.

Second, integrins (ITGs) are adhesion molecules that mediate the connection between the intracellular and extracellular matrix (28). These factors are crucial signaling molecules associated with mechanical and biological signal transduction. The extracellular domain of ITGs can recognize peptides containing the RGD (Arg-Gly-Asp) sequence within the ECM and bind to them, while the cytoplasmic domain of ITGs can connect to the cellular cytoskeleton through linker proteins. The signals generated by the ECM are received by ITGs, and ITG clustering occurs in response to binding to the ECM, thereby initiating intracellular signaling. In mechanically stretched healthy HVFs, the transcription levels of ITGs (ITGA1, ITGA4, ITGAV, and ITGB1) and matrix metalloproteinases (MMP2, MMP8, MMP13) are downregulated, leading to reduced ECM degradation. In stretched POP- HVFs, MMP1, MMP3, MMP10, ADAMTS8, ADAMTS13, TIMP1-3, ITGs (ITGA2, ITGA4, ITGA6, ITGB1), contactin 1 (CNTN1), cadherin A1, cadherin B1, and laminin (LN) were significantly upregulated, while COLs (1, 4–6, 11, 12) and LOXL1 were downregulated. The downregulation of LOX inhibits the promoter activity of COL3A1, leading to impaired collagen synthesis.

**Figure 1:**
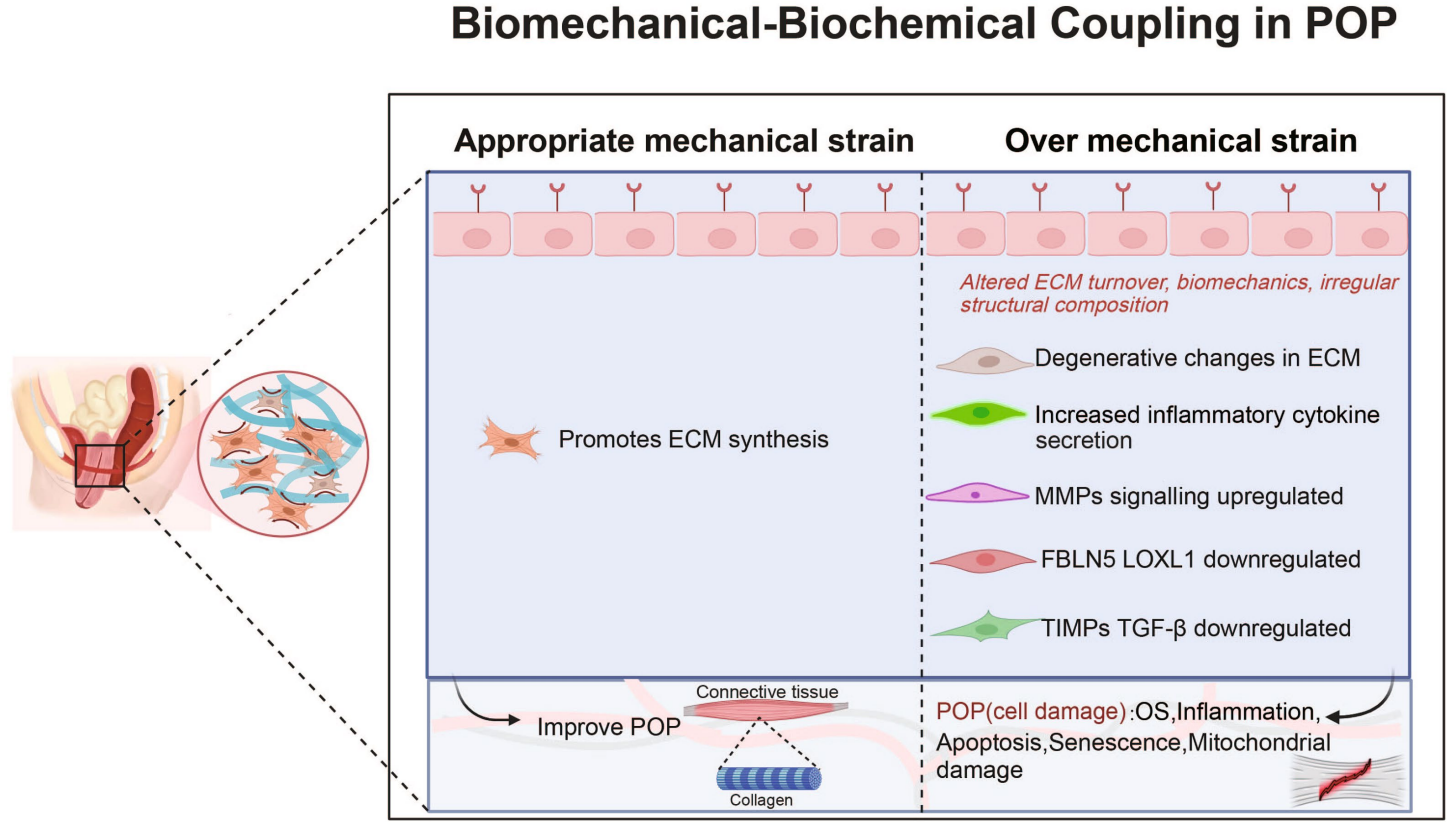
Roles and mechanisms of biomechanical-biochemical coupling in POP. POP, pelvic organ prolapse; MMP, matrix metalloproteinase; OS, Oxidative Stress; FBLN5, fibulin-5; LOXL1, lysyl oxidase like protein 1; TGF-β1, transforming growth factor-β1; TIMP, tissue inhibitors of metalloproteinases.

These factors lead to an increase in ECM degradation and a decrease in collagen synthesis (see Table 1 and Figure 1). Clearly, biomechanical forces, which are risk factors, disrupt the mechanical–chemical microenvironment of POP-HVFs, particularly ECM components and cell-ECM interactions, ultimately resulting in the differential responses of fibroblasts to exogenous mechanical stretching. This weakens the support of pelvic organs and leads to the subsequent development of POP. Fibroblast mechanoreceptor damage promotes POP. Moreover, other studies have demonstrated that cells sense the physical properties of the ECM and activate a mitochondrial stress response (44).

**Table 1:** Association between mechanical stretch and ECM expression in pelvic fibroblasts.

No	Years	Cell	Stretch	Parameter	Method	Control		POP		
						N	Results	N	Grade	Results
1	2010 (32)	HVF	Cyclically biaxially stretched	72 h at 8% or 16% and 1 Hz	Collagenase activity assay	1	↑:Collagenase activity	2	II, III	↑:Collagenase activity
2	2013 (26)	HVF	Cyclic mechanical	48 h at 10% and 0.2 Hz	WB；RT-qPCR	1	↑:MMP (2, 9)/COL1	2	II, IV	↓:COLs (1, 3)
3	2014 (27)	HVF	Cyclic mechanical	48 h at 10% and 0.2 Hz	WB；RT-qPCR	8	↑:MMP14/TIMP1	10	II, III, IV	↑:MMP (2, 14)/TIMP (1, 2)
4	2015 (33)	HVF	Cyclic mechanical	12 h at 10% and 0.1 Hz	IF	5	↑:F-actin	10	III, IV	↓:F-actin
5	2016 (28)	HVF	Mechanical	24 h at 10%	Human ECM and Adhesion Molecules PCR array; WB; RT-qPCR	7	↓:ITGs1 (A1, A4, AV, B1)/MMP (2, 8, 13)	8	III, IV	↑:MMP (1, 3, 10)/ ADAMTS (8, 13)/TIMP (1, 2, 3)/ITGs (A2, A4, A6, B1)/ CNTN1/ catenins (A1, B1)/LNs (A3, C1)；↓: COLs (1, 4, 5, 6, 11, 12)/ LOXL1
6	2018 (29)	HVF	Cyclic mechanical	10% and 0.1 Hz	RT-qPCR	6	↑:COL1/BGN	6	III, IV	↓:DCN/ FMO
7	2008 (34)	hUSLF	Mechanical	96 h at 20%	cDNA microarrays	/	↑:F-actin/ TRADD/ DNase 1-L1/ SIPA-1/MMP20	None	None	None
8	2019 (35)	HVF	Cyclic mechanical	24 h or 72 h at 10% and 0.2 Hz	RT-qPCR	/	/	1	II	↑:COLs (1, 3, 5)/ Elastin /a-SMA/TGF-b1/MMP2/COX-2/TNF-a/IL- 8/IL- 1b
9	2011 (36)	hUSLF	Mechanical	24 h at 4% or 8% or12%	RT-qPCR	8	↑:COLs (1, 3)/ PH/MMP1	None	None	None
10	2016 (37)	hUSLF	Mechanical	0.3 Hz and 5,333 μ	WB	20	↓:COL1	None	None	None
11	2017 (38)	hUSLF	Mechanical	4 h at 0 μ, 1,333 μ, 2,666 μ, 5,333 μ, and 0.1 Hz	IF	10	↑:F-actin	None	None	None
12	2017 (39)	hUSLF	Cyclic mechanical	4 h at 5333 μ and 0.3 Hz	WB; RT-qPCR	/	↑:MMP (2, 9);↓:COLs (1, 3)/Elastin /TIMP2	None	None	None
13	2017 (40)	hUSLF	Cyclic mechanical	4 h at 0 μ, 5,333 μ and 0.1 Hz	WB; RT-qPCR	10	↑:MMP (2, 9);↓: TIMP2/COLs (1, 3)/ Elastin/ TGF-β1	None	None	None
14	2017 (41)	hUSLF	Mechanical	4 h at 0 μ, 1,333 μ, 5,333 μ and 0.5 Hz	WB; RT-qPCR	15	↑:MMP (2, 9); ↓:COLs (1, 3)/Elastin /TGF-β1	None	None	None
15	2017 (42)	hUSLF	Mechanical	4 h at 0 μ, 1,333 μ, 5,333 μ and 0.5 Hz	WB; RT-qPCR	15	↑:MMP2/TIMP2;↓: COLs (1, 3)/ Elastin /TGF-β1	None	None	None
16	2022 (43)	hUSLF	Uniaxial cyclic stress	4 h at 10% and 0.1 Hz	RT-qPCR	8	↑: MMP1; ↓: COLs (1, 3)	10	III, IV	↑: F-actin

POP, pelvic organ prolapse; HVF, human vaginal fibroblast; hUSLF, human uterosacral ligament fibroblasts; IF, Immunofluorescent staining; COL, Collagen type; a-SMA, α-smooth muscle actin; TGF-β1, transforming growth factor-β1; MMP, matrix metalloproteinase; COX, Cyclooxygenase; TNF-a, Tumor Necrosis Factor α; IL, interleukin; TIMP, tissue inhibitors of metalloproteinases; ITG, Integrin; ADAMTS8, a disintegrin like and metalloproteinase with thrombospondin type; CNTN, contactin; LOXL, lysyl oxidase like; BGN, Recombinant Biglycan; DCN, Decorin; FMO, fibromodulin; TRADD, tumor necrosis factor receptor-associated death domain protein; SIPA-1, signal-induced proliferation-associated protein 1; LN, Laminin; /, Not reported.

### 3.2 Mechanobiology mediates cellular remodeling

Mechanical forces activate proteins associated with reconstructing the cytoskeleton in fibroblasts (see Table 1 and Figure 1). The cytoskeleton, which is the largest cellular mechanosensor, serves as the primary mechanism for transmitting mechanical stimuli from the extracellular space to the intracellular compartment. Ruiz-Zapata showed that the cytoskeleton aligns perpendicularly to the force after mechanical stimulation and that the cell reacts to mechanical stimulation by rearranging its F-actin cytoskeleton (26). F-actin is the main skeletal structure that plays an important role in helping the cell withstand external tension and stress. The changes in F-actin in POP-related fibroblasts are evident. Wang et al. showed that under static culture conditions, POP-HVFs exhibited less cell roundness and higher relative fluorescence intensities (RFIs) of cytoskeletal proteins (F-actin, α-tubulin, and waveform protein). However, after stretching, the RFI of the cytoskeleton increased in the normal group, whereas the POP group exhibited a decrease in the RFI of F-actin (33). Thus, the cytoskeleton is remodeled in response to mechanical forces. Similarly, human USL fibroblasts (hUSLFs) not only regulate cellular morphology but also modulate cellular mechanical functions in response to mechanical forces. Among hUSLFs, POP-hUSLFs exhibit larger and longer morphologies, and mechanical stretching results in the dissolution of cytoskeletal proteins. The cytoskeleton undergoes continuous deconstruction and remodeling, which is the first step in mechanical signaling (38). Another study provided the opposite evidence: the mechanical force applied to healthy hUSLFs induces a POP-like change in morphology (43). In the second step, mechanical stretching upregulates genes related to cytoskeletal proteins to regulate cell morphology, including signal-induced proliferation-associated protein 1 (SIPA-1), tumor necrosis factor receptor-associated death domain protein (TRADD), and DNase 1-L1 protein, thereby inhibiting cytoskeletal remodeling, preventing adhesion, and inhibiting actin polymerization. Moreover, MMP20 transcription is upregulated (34). Furthermore, biomechanical force mediates the functional defects in fibroblast through the cellular cytoskeleton, leading to a reduction in mechanical tolerance (33). After the application of mechanical forces, transmission electron microscopy revealed that the polymers in POP-HVFs were stretched strips and fractured mesh. Compared with that of healthy HVFs, the mechanical tolerance of cells was severely degraded (29). Contrary to Wang et al., Zhu et al. discovered that other POP fibroblasts have thicker F-actin stress fibers (43). However, these thickened F-actin stress fibers may put cells in a subtension state, resulting in reduced cell contractility and decreased responsiveness to external stimuli. Additionally, in comparison to those in the normal group, the mRNA levels of type I and III collagen were reduced in stretched POP-HVFs, indicating weakened tissue stiffness and elasticity, decreased biological resilience, and functional defects in fibroblasts. Finally, the cytoskeleton interacts with intracellular proteins and mediates the expression of chemical signals that regulate the ECM response and participate in the functional remodeling of the cell. After excessive mechanical stretching, POP fibroblasts showed downregulated mRNA and protein expression of COL1, COL3, TIMP, elastin and upregulated expression of MMPs, leading to reduced ECM synthesis and a decrease in tissue mechanical properties (40, 41). Similar results have been found in other studies, including the downregulated expression of COL1 and COL3 and upregulated expression of MMPs (43). However, mechanical forces are not just about collagen destruction. In a study of the USL, moderate stress could contribute to pelvic floor collagen synthesis, and too much or too little stress is not conducive to the synthesis of collagen. For example, hUSLFs upregulated COL1 and COL3 expression in response to 8% stress. Interestingly, the effects of COL1 and COL3 on the stress response may be different; the former has a faster reaction than the latter (36). This means that there is still much research to be done on the mechanistic effects on fibroblasts and tissues.

## 4 Impact of biomechanical-biochemical coupling on fibroblast function

### 4.1 Biomechanical induction leads to alterations in the mitochondrial function of fibroblasts

Previous studies have shown that caspase 3 and 9 expression is significantly elevated in the USLs of POP patients, and cytochrome C is released from mitochondria as an upstream activator of caspase 3, which triggers a cellular cascade that leads to cell death, confirming that mitochondrial function is altered and associated with POP (45). Mitochondria play a crucial role in maintaining cellular homeostasis and energy metabolism but are susceptible to damage due to mechanical forces. A normal mitochondrial membrane potential (ΔΨm) indicates mitochondrial health, and a decrease in potential is a hallmark of early apoptosis in cells. HONG et al. demonstrated that when healthy hUSLFs were subjected to 5,333 μ of mechanical stretching, mechanical stress decreased the ΔΨm and induced significant levels of apoptosis in the cells (46). The decrease in ΔΨm activates the mitochondrial apoptotic pathway, leading to cell death. Additionally, mitochondria play a crucial role as regulators of metabolism and are vital for maintaining cell growth and survival. Hu et al. (38) observed that after 4 h of mechanical stress on hUSLFs, cell proliferation decreased significantly, and mitochondrial vacuolization and altered mitochondrial morphology were observed. Furthermore, as mechanical stress increased to a certain level, the cellular structure was disrupted (38). Moreover, mitochondrial dysfunction may initiate senescence. Mechanical force promotes the expression of senescence-associated β-galactosidase (SA β-gal), a marker of cellular aging, thereby accelerating fibroblast senescence (37, 38). As the body ages, mitochondrial biogenesis continues to decline and may affect the transcriptional activity and replication of mitochondrial DNA (47).

### 4.2 Biomechanically induced oxidative stress in fibroblasts

It is suggested that mechanical force can activate the oxidative stress signaling pathway (46). In a study conducted in 2017, it was demonstrated that overexpression of the antioxidant molecule glutathione peroxidase 1 (GPX1) in healthy hUSLFs reversed oxidative stress and restored ΔΨm in response to stretching (40). As a result, the expression of oxidative stress markers such as 8-hydroxy-2-deoxyguanosine (8-OHdG) and 4-hydroxynonenal (4-HNE) decreased, and cell viability remained normal (40). However, in HVFs-induced H2O2-treated cells, oxidative stress led to ECM degradation and a decrease in biomechanical properties (48). Reactive oxygen species (ROS) are the main sources of oxidative stress. ROS accumulation occurs in hUSLFs after mechanical force application, leading to increased apoptosis and cellular senescence in fibroblasts (37, 46). Furthermore, mechanical stress activates the transforming growth factor-β1 (TGF-β1)/Smad signaling pathway, causing an imbalance in the production and degradation of mechanical tissue proteins, which contributes to POP. HONG et al. hypothesized that excessive mechanical stress and H2O2 treatment stimulated oxidative stress, inhibited the phosphorylation of Smad2, activated the TGF-β1/Smad2 signaling pathway, inhibited cell proliferation and remodeled the ECM by downregulating TGF-β1 levels (42). Liu et al. verified a negative correlation between the transcription levels of TGF-β1 and the severity of POP in sacrospinal ligament tissue (39). Conversely, pretreatment of healthy hUSLFs with TGF-β1, followed by cyclic mechanical stretching, improved the phosphorylation of Smad3, activated the TGF-β1/Smad3 signaling pathway, stimulated the synthesis of TIMP-2, inhibited MMP-2/9 activity, reduced ECM loss, and increased ECM synthesis. Notably, the expression of Homeobox11 and TGF-β1 in the sacrospinal ligament of POP patients mediates ECM dysregulation through the regulation of collagen and MMP expression (49), and different types of collagen have been extensively reported to be crucial for maintaining tissue structure and biomechanical function (50), but their effects at the cellular level have not been reported or validated. On the other hand, other studies have shown that activating transcription factor 3 (ATF3) can downregulate ROS through the p38/Nrf2 signaling pathway, protecting vaginal fibroblasts from injury and reducing cell apoptosis. However, oxidative stress is not always harmful; low levels of oxidative stress are beneficial for fibroblasts. In pulmonary fibroblasts, low levels of ROS activate fibroblast proliferation by suppressing antioxidant enzyme stimulation (51). Additionally, TGF-β1 may counteract oxidative stress and promote normal metabolism in normal fibroblasts (42).

### 4.3 Mechanical induction of the fibroblast inflammatory response

Inflammatory responses can generate various soluble inflammatory mediators, and interferon γ (IFN-γ) is a potent proinflammatory molecule. Previous studies have shown that compared to those of non-POP patients, the mRNA levels of IFN-γ and its receptor in the vaginal wall in POP patients were higher, and the mRNA level of nuclear factor-κB (NF-κB) increased by 5.1-fold (52). Miao et al. showed that inflammation in POP is age-related, and there is are increases in biological processes associated with chronic inflammation in older POP patients, while in younger POP patients, these processes are associated with ECM metabolism and possibly related to immune regulation (19, 53). Increased inflammatory responses have also been observed in the USL of POP patients, leading to the definition of inflammatory-type POP at the pathological level (54). However, examination at the cellular level is still lacking. Moreover, mechanical force-induced mitochondrial dysfunction can activate inflammatory responses. When mitochondrial DNA is subjected to high-intensity stress, oxidative stress reactions are activated, leading to the leakage of mitochondrial ROS into the cytoplasm and the excessive activation of inflammatory mediators (55), such as NF-κB, tumor necrosis factor α (TNF-α), nucleotide-binding oligomerization domain-like receptor protein 3 (NLRP3) inflammasomes, and activator protein 1 (AP-1), ultimately affecting the fate of fibroblasts (55). Unfortunately, relevant studies on POP patient cells have not shown crosstalk among the mechanochemical signaling pathways.

### 4.4 Mechanical induction of fibroblast apoptosis

Mechanical force is an environmental factor that affects the survival of pelvic floor cells. Previous proteomics analysis revealed that many differentially expressed proteins in the USLs of POP patients are apoptosis-related proteins (56). At the cellular level, the POP-hUSLF group showed high expression of proapoptotic proteins such as Bad, Bax, and Caspase 3, while the antiapoptotic protein Bcl-2 was decreased (43, 57). Consequently, cell apoptosis was significantly increased, indicating that mechanical force induces cell apoptosis. Overall, these studies suggest that cell apoptosis is a key mechanism underlying POP development. The growth rate of POP-hUSLFs was slower than that of healthy hUSLFs, and their proliferative capacity was impaired (58). Similarly, multiple studies have demonstrated that excessive mechanical stress inhibits cell proliferation and promotes apoptosis through the PI3K/Akt, p38/MAPK, and actin cytoskeleton/Nr4a1 signaling pathways, and estrogen has a protective effect (36, 43, 46, 57, 59). (1) Akt, which is a component of the kinase network, participates in mechanotransduction (60). In healthy hUSLFs, mechanical strain activates the PI3K/Akt signaling pathway: mechanical stimulation rapidly activates Akt, recruiting it from the cytoplasm to the plasma membrane, where it is phosphorylated at Thr308 and Ser473. Activated Akt phosphorylates downstream FOXO1, causing nuclear exclusion of FOXO1 and reducing its ability to regulate target genes, including antioxidant enzyme-encoding genes that protect cells from oxidative damage (61). This leads to the downregulation of GPX1 and manganese superoxide dismutase (Mn-SOD) expression, activation of the oxidative stress signaling pathway, an increase in ROS, the promotion of hUSLF apoptosis and senescence, a decrease in COL1 production, and ultimately cellular depletion, pelvic floor laxity, and functional impairment. A study in 2017 (40) demonstrated that overexpression of GPX1 in healthy hUSLFs and subsequent stretching increased mitochondrial membrane potential, decreased expression of oxidative stress markers (8-OHdG and 4-HNE), inhibited mitochondrial damage, attenuated cell apoptosis, and reduced collagen damage, indicating a protective effect on ECM remodeling in response to mechanical stretching in cells. (2) MAPK is a protein kinase family and a key signaling pathway through which mechanical signals promote cell apoptosis via phosphorylation cascades involving ERK, p38, and JNK. In fibroblasts in the periodontal ligament, activation of the p38/MAPK pathway by mechanical stress has been widely reported (62). Zhu et al. first reported the involvement of the p38/MAPK pathway in mechanotransduction in hUSLFs in the context of POP development (43, 63). After 24 h of stretching, the level of phosphorylated p38 protein was upregulated in healthy hUSLFs, and the increase in phosphorylated p38 was associated with increased expression of the apoptosis genes *Bad* and *Bax*, which could induce *Bax* translocation and increase cell apoptosis. However, there were no differences observed in ERK/MAPK or JNK/MAPK, suggesting that they may not be involved in POP development. It is also possible that the activation of these two pathways is delayed by the duration and intensity of mechanical force. Furthermore, role of the p38/MAPK signaling pathway can be validated by inhibiting or silencing p38. The failure of USL cells to resist excessive mechanical damage may also be a mechanism underlying the development of POP. (3) The actin cytoskeleton/Nr4a1 signaling pathway is involved in mechanical regulation. The actin cytoskeleton determines cell morphology and strength and participates in the transmission of mechanical signals. Nuclear receptor subfamily 4 group A member 1 (Nr4a1) induces apoptosis through its interaction with Bcl-2. In hUSLFs, mechanical stretching can induce disassembly of the actin cytoskeleton. This leads to an increase in Nr4a1 levels through activation and induction. Nr4a1 then regulates the expression of the proapoptotic proteins Bax and Caspase 3 and downregulates the expression of the antiapoptotic protein Bcl-2, thereby promoting cell apoptosis. Knocking down Nr4a1 can reverse the proapoptotic effect induced by stretching. Therefore, the actin cytoskeleton/Nr4a1 pathway is an important mechanism that regulates cell apoptosis during POP development. (4) Estrogen/poly-ADP-ribose polymerase 1 PARP1 reverses mechanical transduction damage. PARP1 is a multifunctional nuclear enzyme that catalyzes the transfer of ADP-ribose units from NAD+ to specific target proteins, which controls important physiological processes such as DNA damage and gene expression (64). PARP1 is an important member of the antiapoptotic protein family. Studies have indicated that mechanical stretching induces cell apoptosis and death in patients and healthy hUSLFs and that poststretching intervention with estrogen improves cell status (58). The estrogen/PARP1 signaling pathway is involved in the development of POP. Estrogen upregulates the expression of the antiapoptotic protein Bcl-2, downregulates the expression of the proapoptotic protein Bax, and enhances expression of the estrogen receptor (ERα). ERα targets poly-ADP-ribosylation to upregulate PARP1, thereby reducing mechanical strain-induced apoptosis and death in hUSLFs. Interestingly, estrogen exhibits this protective effect only when mechanical stretching stimulates PARP expression. Therefore, further research is needed to elucidate the molecular mechanisms underlying this phenomenon.

### 4.5 Mechanical strain with other potential elements

Firstly, Mechanical strain often directly regulate gene expression. When mechanical stretch was applied, normal hUSLs exhibited ROS, apoptosis, and senescence (65). *In vitro* experiments had shown that overexpression of FBLN5 could alleviate mechanical strain-induced damage in hUSLF cells by inhibiting FOSL1 expression. However, knocking out FBLN5 could promote the occurrence of pelvic organ prolapse in the PFD model rats by regulating the FOSL1/miR-222/MEIS1/COL3A1 axis (65). Secondly, lack of effective mechanical strain modeling at present. Using a physiologically relevant 3D *in vitro* model, under static conditions, 3D cultured POP fibroblasts are less proliferative, exhibit lower collagen and elastin contents compared to non-POP fibroblasts. However, under mechanical loading, the differences between POP and non-POP fibroblasts are less pronounced (66). This suggests that although mechanical stress plays a major role, there is no more comprehensive model that accurately simulates the pathophysiology of POP. Meanwhile, *in vivo*, a study first measures whether there is a significant difference in viscohyperelastic behavior of the USL between women with and without POP with a computer-controlled linear servo-actuator and did not differ significantly. Thus, larger sample sizes would help improve the precision of intergroup differences. So that, the study suggests that expanding larger sample sizes would help improve the precision of intergroup differences (67).

## 5 Conclusion

This study presents a comprehensive overview of the biomechanical structure of the anterior vaginal wall and USLs, as well as the interaction between fibroblast biomechanics and chemical signaling. Although significant progress has been made in understanding the biomechanical dysfunction underlying POP, there are still unresolved issues. The details of collagen synthesis, assembly, maturation, and mechanical signal transduction remain unclear, making it challenging to determine whether the observed results are the cause or consequence of prolapse. Additionally, the effects of appropriate force and oxidative stress on fibroblast proliferation and function contradict the findings of earlier studies, calling for further investigation. Furthermore, molecular differences between prolapsed and nonprolapsed sites within the same patient pose challenges in terms of sample collection and research methodologies. Therefore, a systematic compilation of existing research is crucial in guiding future investigations. Moreover, the use of suitable animal and cell models has become increasingly important for gaining deeper insights into the pathogenesis of POP. Ultimately, addressing these issues will contribute to improving the incidence rate of POP, but it requires long-term exploration and research.

## Author contributions

HW: Conceptualization, Writing – original draft. LZ: Supervision, Writing – original draft. LH: Project administration, Writing – review & editing. WL: Project administration, Writing – review & editing. BY: Project administration, Writing – review & editing. XY: Supervision, Writing – review & editing. YL: Conceptualization, Supervision, Writing – original draft.
